# Role of Antipseudomonal Antibiotics in Older Patients with Aspiration Pneumonia: A Nationwide Database Study in Japan

**DOI:** 10.3390/antibiotics14080743

**Published:** 2025-07-24

**Authors:** Ryohei Kudoh, Daisuke Yoneoka, Akihiko Hagiwara, Hisayuki Shuto, Shota Omori, Kiyohide Fushimi, Kosaku Komiya

**Affiliations:** 1 Respiratory Medicine and Infectious Diseases, Oita University Faculty of Medicine, 1-1 Idaigaoka, Hasama-machi, Yufu 879-5593, Oita, Japan; 2 Department of Epidemiology, National Institute of Infectious Diseases, Japan Institute for Health Security, Tokyo 162-8640, Japan; 3 Department of Health Policy and Informatics, Institute of Science Tokyo, 1-5-45 Yushima, Bunkyo-ku, Tokyo 113-8519, Japan

**Keywords:** aspiration pneumonia, antipseudomonal antibiotics, older patients, in-hospital mortality

## Abstract

**Background**: Aspiration pneumonia is increasingly recognized as a fatal pulmonary disease among older people. Although antipseudomonal antibiotics are commonly used in clinical practice, their efficacy in this population remains uncertain. **Methods**: Nationwide data collected from patients aged ≥65 years who were hospitalized due to aspiration pneumonia from January 2018 to December 2018 were analyzed. The in-hospital mortality between patients who received antipseudomonal antibiotics within 3 days of hospital admission and those who did not were compared. A logistic regression analysis was performed to assess the effect of antipseudomonal antibiotics on in-hospital mortality after adjusting for potential prognostic confounders. **Results**: This study included 46,980 patients, and 13,340 (28.4%) patients received antipseudomonal antibiotics. In total, 7011 (14.9%) patients died during hospitalization. Advanced age, male sex, a lower body mass index, decreased Barthel Index, impaired consciousness, interstitial pneumonia, malignancy, renal failure, and use of immunosuppressive agents were significantly associated with increased in-hospital mortality. After adjusting for the confounders, the use of antipseudomonal antibiotics was found to be associated with an elevated in-hospital mortality (odds ratio: 1.33; 95% confidence interval: 1.26–1.41; *p* < 0.001). **Conclusions**: In this nationwide data analysis of older patients with aspiration pneumonia, early antipseudomonal antibiotic administration did not improve prognosis. Therefore, the routine use of antipseudomonal antibiotics should be avoided in older patients with aspiration pneumonia.

## 1. Introduction

Aspiration pneumonia is defined as pneumonia caused by the aspiration of oropharyngeal or upper gastrointestinal contents, commonly due to decreased swallowing function [1,2]. Age-related physiological changes, including general frailty, residual cerebrovascular diseases, prolonged immobilization, and malnutrition are risk factors for the development of aspiration pneumonia [3,4,5]. The proportion of patients with aspiration pneumonia increased with age in hospitalized patients with pneumonia in Japan [6], and 89% of patients who died from aspiration pneumonia were aged >65 years [7]. The morbidity and mortality rates of aspiration pneumonia continue to increase in regions with aging populations [8].

Previous studies have reported about prognostic factors such as advanced age and impaired functional status [9,10,11]. However, evidence guiding empirical antibiotic selection in this population is limited. For example, empirical coverage for *Pseudomonas aeruginosa* was traditionally recommended for patients with risk factors for healthcare-associated infections or severe diseases [12], but recent data suggest that this practice may not improve outcomes in older adults. In a systematic review examining antibiotic treatment in older patients with aspiration pneumonia, no clinical trials demonstrated superior efficacy of broad-spectrum or antipseudomonal agents—such as piperacillin–tazobactam, cefepime, meropenem, or ciprofloxacin. Moreover, their use was associated with an increased risk of multidrug-resistant organism emergence [13]. Similarly, a retrospective cohort of elderly patients with pneumonia and *P. aeruginosa* isolation revealed that the use of antipseudomonal antibiotics did not reduce 28-day mortality [14]. Such studies are limited, and there is no nationwide evidence supporting the abovementioned notion. Nevertheless, the updated guidelines of the American Thoracic Society and Infectious Diseases Society of America (2019) no longer recommend routine antipseudomonal coverage for all patients with risk factors for multidrug resistance [15].

Considering the revised concept of empirical antibiotic treatment options for older patients with aspiration pneumonia in the guidelines based on insufficient high-quality evidence, it is important to gather more reliable evidence on the use of early antipseudomonal antibiotics in these patients. However, a sufficiently large analysis focusing on the role of broad-spectrum antibiotics on patient prognosis was not performed. To address this gap, a nationwide cohort study was performed using the Japanese Diagnosis Procedure Combination (DPC) database, which provides data on most hospitalizations caused by acute illnesses in Japan [16]. This study aimed to assess the impact of the early use of antipseudomonal antibiotics on in-hospital mortality among patients aged ≥65 years who presented with aspiration pneumonia.

## 2. Results

**Figure 1:**
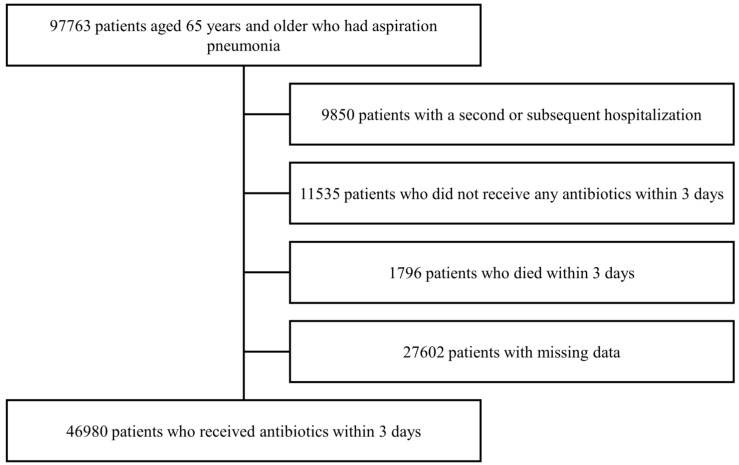
Flow chart of the patient selection process.

Data on 97,763 patients aged ≥65 years who were hospitalized due to aspiration pneumonia from January 2018 to December 2018 were extracted from the DPC database. After excluding patients with a second or subsequent hospitalization during the study period, those who did not receive antibiotic therapy within 3 days after hospital admission, those who died within 3 days of hospitalization, or those with missing data, 46,980 were included in this study (Figure 1). The mean age of the overall cohort was 85.2 years, and 25,284 (53.8%) patients were men. The mean body mass index (BMI) was 19.1 kg/m^2^, and the mean Barthel Index was 17.5. Approximately 51.6% (*n* = 24,220) of the patients required emergency transportation. Further, 66.6% (*n* = 31,292) of the patients received oxygen therapy. However, only 1282 (2.7%) of the patients were managed with mechanical ventilation. In total, 7011 (14.9%) of the patients died during hospitalization, and 13,340 (28.4%) of the patients received antipseudomonal antibiotics.

Compared with survivors, non-survivors were older, more likely to be men, had a lower BMI and lower Barthel index, and were more frequently associated with a history of smoking, impaired consciousness, emergency transportation, and a higher severity based on the Hugh–Jones classification (Table 1). The non-survivor group was more likely to present with comorbidities at admission, including chronic heart failure, interstitial pneumonia, chronic obstructive pulmonary disease (COPD), bronchiectasis, renal failure, and malignant tumors, than the survivor group. As for in-hospital interventions, the use of systemic steroids or immunosuppressants, treatment with antipseudomonal antibiotics, oxygen administration, and mechanical ventilation were more common in the non-survivor group than in the survivor group.

According to the univariate logistic regression analyses, several factors, including baseline characteristics of the patients and comorbidities, were significantly associated with in-hospital mortality (Table 2). The use of antipseudomonal antibiotics was negatively associated with survival (odds ratio: 1.502, *p* < 0.001). In the multivariate analysis, older age, male sex, and a lower BMI and Barthel Index were significantly associated with increased in-hospital mortality (Table 3). Other factors, such as impaired consciousness, chronic heart failure, interstitial pneumonia, COPD, bronchiectasis, renal failure, malignancy, and the use of steroids or immunosuppressants, were also associated with mortality. After adjusting for the characteristics of the patients and disease severity, the use of antipseudomonal antibiotics was not associated with reduced in-hospital mortality. However, it was still associated with increased mortality (odds ratio: 1.333, *p* < 0.001). To further investigate this association, we conducted additional multivariate analyses stratified by the subclass of antipseudomonal antibiotics. The use of carbapenems (Supplementary Table S1) and non-carbapenem antipseudomonal agents (Supplementary Table S2) was independently associated with increased in-hospital mortality (odds ratios: 1.487 and 1.217, respectively; both *p* < 0.001), indicating that this trend was consistent across antibiotic subclasses. Conversely, dementia, asthma, and cerebrovascular disease were associated with decreased odds of in-hospital mortality.

## 3. Discussion

In this nationwide cohort study including 46,980 older patients who were hospitalized due to aspiration pneumonia, the use of antipseudomonal antibiotics was independently associated with increased in-hospital mortality even after adjusting for the baseline characteristics of patients and disease severity, thereby indicating that their empirical use does not improve outcomes in this population. Advanced age, male sex, a lower BMI, decreased Barthel Index, impaired consciousness, chronic heart failure, interstitial pneumonia, COPD, bronchiectasis, renal failure, malignancy, and the use of systemic steroids or immunosuppressants were significantly associated with increased mortality. However, some comorbidities, including dementia, asthma, and cerebrovascular disease, were also associated with decreased odds of in-hospital mortality.

Considering that there are no randomized controlled trials on the efficacy of broad-spectrum antibiotic treatment among older patients with pneumonia, evidence showing that the use of antipseudomonal antibiotics was independently associated with higher in-hospital mortality indicates that the routine administration of broad-spectrum antibiotics covering *P. aeruginosa* may not confirm survival benefits in these patients. Although this study could not evaluate the causes for the adverse effects of antipseudomonal antibiotics on survival, a retrospective analysis conducted in four emergency departments in the USA including patient data obtained in 1995 showed that the use of antipseudomonal agents in CAP was associated with a significantly higher 30-day mortality rate, increased risk of *Clostridioides difficile* infection, and prolonged hospitalization [17]. The adverse effects of broad-spectrum antibiotic treatments might be related to higher in-hospital mortality among older patients.

Furthermore, even if *P. aeruginosa* is truly the causative pathogen, if antipseudomonal antibiotics may microbiologically kill the bacteria, the effects might be clinically limited due to vulnerable host factors, such as advanced age [9], low serum albumin [10], and low muscle mass [11]. In fact, a recent systematic review of factors associated with poor prognosis despite appropriate antibiotic usage for pneumonia has revealed that advanced age and decreased physical activity levels can be associated with increased mortality [18]. Although no specific antibiotic, even antipseudomonal medications, had superior clinical efficacy, their use was associated with the emergence of multidrug-resistant organisms [13]. Given the low prevalence of *P. aeruginosa* in aspiration pneumonia and the possible adverse effects of broad-spectrum antibiotic exposure, our results indicate the need for more judicious selection of empiric antibiotics in older patients. Wijit et al. proposed a model that can predict *P. aeruginosa* infection in older adults with community-acquired pneumonia based on factors such as bronchiectasis, underlying pulmonary disease, immunocompromised status, and enteral nasogastric tube feeding [19]. Such tools might provide guidance on the more appropriate use of antipseudomonal antibiotics. Moreover, a previous study reported that a substantial proportion of pneumonia cases improved without antipseudomonal therapy, even when *Pseudomonas aeruginosa* was isolated from respiratory specimens [14]. This finding implies that *P. aeruginosa* may sometimes reflect colonization rather than true infection, reinforcing the view that antipseudomonal antibiotics are not invariably associated with improved clinical outcomes.

Advanced age, male sex, a lower BMI, decreased Barthel Index scores, impaired consciousness, chronic heart failure, interstitial pneumonia, COPD, bronchiectasis, renal failure, malignancy, and the use of systemic steroids or immunosuppressants were significantly associated with increased mortality. These findings are consistent with those of previous studies. For example, a previous report has shown that older age and male sex were associated with higher mortality rates in elderly patients with CAP [20]. A lower BMI has been consistently associated with increased mortality risk, potentially due to decreased physiological reserves and nutritional deficiencies that impair immune response [21]. The association between decreased Barthel Index scores and a higher mortality reflects the impact of functional status on patient outcomes. This finding is consistent with that of the study conducted by Yoshimatsu et al., who showed that frailty and functional decline are key contributors to poor prognosis in older adults with pneumonia [22]. The increased mortality associated with comorbid conditions such as chronic heart failure, interstitial pneumonia, COPD, bronchiectasis, renal failure, and malignancy is associated with previous findings in the literature. Lanspa et al. [23] revealed that aspiration pneumonia is frequently associated with poor outcomes, particularly among patients with underlying chronic cardiopulmonary diseases, contributing to a 30-day mortality rate of >20%. Another study showed that COPD and chronic kidney disease are significant predictors of mortality in patients with dysphagia, a major risk factor for aspiration [24].

These findings emphasize the importance of accounting for comorbidities in prognostic assessment and treatment decision-making for aspiration pneumonia. Meanwhile, our study found that dementia, asthma, and cerebrovascular disease were associated with decreased odds of in-hospital mortality. This contrasts with some previous studies showing that dementia is a risk factor for increased mortality in aspiration pneumonia [25]. The discrepancy may be attributed to differences in study populations, healthcare settings, or management approaches. Nevertheless, further research should be conducted to elucidate the protective factors that may contribute to improved outcomes in these patient subsets.

A major strength of this study is that it performed a large-scale analysis using data from a nationwide database, which enhances the generalizability of our findings to the older population who were hospitalized because of pneumonia attributed to aspiration. However, it also has some limitations that should be acknowledged. First, the retrospective cohort design might have introduced selection bias. Antipseudomonal antibiotics are generally selected based on the risk factors of drug-resistant bacterial infection and disease severity. We adjusted the impact of antipseudomonal antibiotic treatments with the baseline characteristics of the patients, comorbidities, and clinical factors that were potentially associated with disease severity. Nevertheless, other undocumented factors, such as the beliefs of attending physicians, may affect decision-making in antibiotic selection. Moreover, due to the lack of certain clinical information in the DPC data, adjustment for disease severity may have been insufficient. As the available variables did not adequately predict the use of antipseudomonal antibiotics, propensity score matching was not feasible. Second, the study was based on administrative claims data. However, detailed clinical information, such as laboratory findings, imaging data, and microbiological test results, could not be obtained. Thus, antipseudomonal antibiotics were not necessarily administered to patients with sputum samples containing *P. aeruginosa*. Third, the current study does not account for variations in treatment protocols, including dose, route of administration, and treatment period, across different institutions, which may influence patient outcomes.

## 4. Materials and Methods

### 4.1. Data Source

This study used national datasets obtained from the DPC database, which is a nationwide inpatient database in Japan. This database is linked to a lump-sum payment system for inpatients in >1600 acute-care hospitals [26]. The dataset includes the following details: age, sex, height and weight, diagnosis, comorbidities upon admission that were coded with the International Classification of Diseases 10th Revision (ICD-10) codes, smoking history, Hugh–Jones classification, state of consciousness that was evaluated using the Japan Coma Scale, Barthel Index, emergency transport, medical procedures, use of medications, length of hospital stay, and discharge status. The Institutional Ethics Committee of the Oita University Faculty of Medicine approved this study (approval no. 2764, approval date: 22 March 2024). The study was performed in accordance with the Declaration of Helsinki. The requirement for informed consent was waived by the ethics committee due to the retrospective nature of the study. Information on this research was posted at the hospital.

### 4.2. Study Population

To exclude bias associated with coronavirus disease 2019, this study included patients ≥ 65 years who were hospitalized due to aspiration pneumonia (ICD-10 code J690) from January 2018 to December 2018. Patients with a second or subsequent hospitalization during the study period, those with no history of antibiotic therapy within 3 days, those who died within 3 days, or those with missing data were excluded from the analysis.

### 4.3. Data Collection

Data on the characteristics of the patients upon admission, including age, sex, height, weight, Barthel Index, smoking history, Japan Coma Scale score, emergency transport, and Hugh–Jones classification, were collected. Details on comorbidities at admission, including chronic heart failure, dementia, interstitial pneumonia, asthma, chronic obstructive pulmonary disease (COPD), bronchiectasis, collagen disease, liver failure, diabetes, hemiplegia or paraplegia, renal failure, malignancy, acquired immunodeficiency syndrome/human immunodeficiency virus, and cerebrovascular disease, were collected using the Charlson Comorbidity Index [27,28], as a reference. Data on the medications administered during hospitalization were also collected. The antibiotics were based on those listed in the community-acquired pneumonia (CAP), nursing and healthcare associated pneumonia, and hospital-acquired pneumonia sections of the Japanese Respiratory Society Guidelines for the Management of Pneumonia in Adults 2017 [29]. In particular, the medications included penicillins, such as sulbactam/ampicillin, piperacillin (PIPC, tazobactam/PIPC); cephalosporins, such as ceftriaxone, cefotaxime, ceftazidime, cefepime, cefozopran, and cefpirome; macrolides, such as azithromycin; carbapenems, such as imipenem/cilastatin, meropenem, doripenem, and biapenem; quinolones, such as levofloxacin, ciprofloxacin, and pazufloxacin mesylate; tetracyclines, such as minocycline; oxazolidinones such as linezolid; glycopeptides, such as vancomycin and teicoplanin; aminoglycosides, such as gentamicin, amikacin, tobramycin, and arbekacin; lincomycins, such as clindamycin; and nitroimidazoles, such as metronidazole. The following agents were defined as antipseudomonal antibiotics: PIPC, tazobactam/PIPC, ceftazidime, cefepime, cefozopran, cefpirome, imipenem/cilastatin, meropenem, doripenem, biapenem, levofloxacin, ciprofloxacin, pazufloxacin mesylate, gentamicin, amikacin, and tobramycin. Immunosuppressive agents were defined according to the World Health Organization classification [30]. Only those approved for reimbursement under the Japanese national health insurance system were included. In addition, data on in-hospital interventions such as oxygen administration and mechanical ventilation were collected.

### 4.4. Statistical Analysis

Statistical analyses were performed using the Statistical Package for the Social Sciences version 22 (IBM, Armonk, NY, USA). The characteristics of the patients and medical interventions during hospitalization were compared between the non-survivor and survivor groups using the independent samples *t*-test for continuous variables and the chi-square (χ^2^) test or Fisher’s exact test for categorical variables. *p*-values > 0.05 indicated statistically significant differences. A multivariable logistic regression analysis was performed to assess the effect of antipseudomonal antibiotic use on in-hospital mortality after adjusting for confounders.

## 5. Conclusions

This nationwide retrospective cohort study showed that the use of antipseudomonal antibiotics within the first 3 days of hospital admission was associated with increased in-hospital mortality in older patients with aspiration pneumonia, even after adjusting for the characteristics of the patients, comorbidities, and clinical severity. These findings indicate that the empirical administration of antipseudomonal agents does not confer survival benefit in this population. Hence, physicians should avoid the routine use of antipseudomonal antibiotics in older patients with aspiration pneumonia. However, the results are based on an observational study, which may have inherent biases. Therefore, a randomized controlled interventional trial should be performed to validate our results. Moreover, further studies must be performed to identify which patient populations may benefit from treatments with broad-spectrum antibiotics.

## Figures and Tables

**Table 1:** Clinical baseline characteristics of patients.

	Survivors (*n* = 39,969)	Non-Survivors (*n* = 7011)	*p* Value
Age (years)	85.0 ± 7.8	86.2 ± 7.4	<0.001
Sex (male)	21,035 (52.6)	4249 (60.6)	<0.001
BMI (kg/m^2^)	19.3 ± 3.8	18.1 ± 3.6	<0.001
Barthel Index	18.9 ± 31.5	9.1 ± 22.2	<0.001
Current/past smoker	9297 (23.3)	1750 (25.0)	0.002
Coma	1423 (3.6)	516 (7.4)	<0.001
Emergency transport	20,219 (50.6)	4001 (57.1)	<0.001
Hugh–Jones classification			<0.001
1	2137 (5.3)	123 (1.8)	
2	2324 (5.8)	144 (2.1)	
3	2264 (5.7)	164 (2.3)	
4	4909 (12.3)	449 (6.4)	
5	6809 (17.0)	1626 (23.2)	
Unclassifiable	21,526 (53.9)	4505 (64.3)	
Chronic heart failure	5632 (14.1)	1363 (19.4)	<0.001
Dementia	11,586 (29.0)	1902 (27.1)	0.002
Interstitial pneumonia	432 (1.1)	147 (2.1)	<0.001
Asthma	2017 (5.0)	292 (4.2)	0.002
COPD	1035 (2.6)	220 (3.1)	0.009
Bronchiectasis	339 (0.8)	182 (1.2)	0.008
Collagen disease	609 (1.5)	96 (1.4)	0.327
Liver failure	677 (1.7)	116 (1.7)	0.814
Diabetes	6107 (15.3)	1039 (14.8)	0.323
Hemiplegia and paraplegia	148 (0.4)	25 (0.4)	0.861
Renal failure	2141 (5.4)	545 (7.8)	<0.001
Malignancy	3341 (8.4)	857 (12.2)	<0.001
AIDS/HIV	2 (<0.1)	0 (0)	1.000
Cerebrovascular disease	9217 (23.1)	1451 (20.7)	<0.001
Steroids or immunosuppressants	2981 (7.5)	744 (10.6)	<0.001
Coverage of *P. aeruginosa*	10,829 (27.1)	2511 (35.8)	<0.001
Use of carbapenem	1903 (4.8)	592 (8.4)	<0.001
Use of non-carbapenem antipseudomonal antibiotics	8926 (22.3)	1919 (27.4)	<0.001
Oxygen administration	25,712 (64.3)	5580 (79.6)	<0.001
Mechanical ventilation	896 (2.2)	386 (5.5)	<0.001

Values are presented as the means ± standard deviation or number (%). Abbreviations: AIDS, acquired immunodeficiency syndrome; BMI, body mass index; COPD, chronic obstructive pulmonary disease; HIV, human immunodeficiency virus.

**Table 2:** Univariate analysis of factors associated with in-hospital mortality.

	Odds Ratio	95% CI	*p* Value
Age (years)	1.021	1.018–1.024	<0.001
Sex (male)	1.385	1.315–1.458	<0.001
BMI (kg/m^2^)	0.909	0.902–0.916	<0.001
Barthel Index	0.986	0.985–0.987	<0.001
Current/past smoker	1.097	1.035–1.164	0.002
Coma	2.152	1.939–2.388	<0.001
Emergency transport	1.298	1.234–1.367	<0.001
Hugh–Jones classification	0.968	0.956–0.980	<0.001
Chronic heart failure	1.471	1.378–1.571	<0.001
Dementia	0.912	0.862–0.965	0.002
Interstitial pneumonia	1.960	1.623–2.368	<0.001
Asthma	0.818	0.721–0.927	0.002
COPD	1.219	1.051–1.413	0.009
Bronchiectasis	1.383	1.086–1.763	0.008
Collagen disease	0.897	0.722–1.114	0.327
Liver failure	0.976	0.801–1.191	0.814
Diabetes	0.965	0.898–1.036	0.323
Hemiplegia and paraplegia	0.963	0.630–1.472	0.861
Renal failure	1.489	1.351–1.642	<0.001
Malignancy	1.527	1.410–1.653	<0.001
AIDS/HIV	–	–	1.000
Cerebrovascular disease	0.871	0.818–0.927	<0.001
Steroids or immunosuppressants	1.473	1.353–1.603	<0.001
Coverage of *P. aeruginosa*	1.502	1.423–1.584	<0.001
Use of carbapenem	1.845	1.676–2.031	<0.001
Use of non-carbapenem antipseudomonal antibiotics	1.311	1.237–1.388	<0.001
Oxygen administration	2.162	2.033–2.299	<0.001
Mechanical ventilation	2.541	2.249–2.871	<0.001

Abbreviations: AIDS, acquired immunodeficiency syndrome; BMI, body mass index; CI, confidence interval; COPD, chronic obstructive pulmonary disease; HIV, human immunodeficiency virus.

**Table 3:** Multivariate analysis of factors associated with in-hospital mortality (including variables with significance in univariate analysis).

	Odds Ratio	95% CI	*p* Value
Age (years)	1.027	1.024–1.031	<0.001
Sex (male)	1.733	1.631–1.842	<0.001
BMI (kg/m^2^)	0.911	0.904–0.918	<0.001
Barthel Index	0.987	0.986–0.989	<0.001
Current/past smoker	0.976	0.912–1.046	0.495
Coma	1.672	1.497–1.867	<0.001
Emergency transport	0.954	0.902–1.009	0.097
Hugh–Jones classification	0.989	0.976–1.002	0.086
Chronic heart failure	1.400	1.306–1.501	<0.001
Dementia	0.868	0.818–0.922	<0.001
Interstitial pneumonia	2.033	1.661–2.488	<0.001
Asthma	0.688	0.580–0.816	<0.001
COPD	1.349	1.101–1.654	0.004
Bronchiectasis	1.517	1.175–1.959	0.001
Renal failure	1.535	1.385–1.702	<0.001
Malignancy	1.764	1.618–1.922	<0.001
Cerebrovascular disease	0.867	0.812–0.925	<0.001
Steroids or immunosuppressants	1.390	1.269–1.523	<0.001
Coverage of *P. aeruginosa*	1.333	1.261–1.410	<0.001
Oxygen administration	1.857	1.737–1.985	<0.001
Mechanical ventilation	1.658	1.454–1.891	<0.001

Abbreviations: BMI, body mass index; CI, confidence interval; COPD, chronic obstructive pulmonary disease.

## Data Availability

The data that support the findings of this study are available from the corresponding author upon reasonable request.

## Supplementary Materials

The following supporting information can be downloaded at: https://www.mdpi.com/article/10.3390/antibiotics14080743/s1, Table S1. Multivariate analysis of factors associated with in-hospital mortality: use of carbapenems. Table S2. Multivariate analysis of factors associated with in-hospital mortality: use of non-carbapenem antipseudomonal antibiotics.
