# Supplementary material for: Role of Antipseudomonal Antibiotics in Older Patients with Aspiration Pneumonia: A Nationwide Database Study in Japan
Source: Antibiotics (Basel). 2025 Jul 24;14(8):743. doi: 10.3390/antibiotics14080743 (PMC12382901; doi:10.3390/antibiotics14080743)
Supplement: Supplementary file 1 [file antibiotics-14-00743-s001.zip › antibiotics-3758317-supplementary.pdf]

Table S1. Multivariate analysis of factors associated with in-hospital mortality: use of carbapenems

	Odds ratio	95% CI	*p* value
Age (years)	1.027	1.023-1.031	<0.001
Sex (male)	1.743	1.640-1.852	<0.001
BMI (kg/m^2)	0.910	0.903-0.918	<0.001
Barthel index	0.987	0.986-0.989	<0.001
Current/past smoker	0.973	0.909-1.042	0.436
Coma	1.672	1.496-1.867	<0.001
Emergency transport	0.951	0.900-1.006	0.082
Hugh-jones classification	0.989	0.977-1.002	0.104
Chronic heart failure	1.399	1.305-1.500	<0.001
Dementia	0.874	0.823-0.928	<0.001
Interstitial pneumonia	2.054	1.679-2.514	<0.001
Asthma	0.691	0.582-0.819	<0.001
COPD	1.338	1.091-1.640	0.005
Bronchiectasis	1.531	1.185-1.977	0.001
Renal failure	1.531	1.381-1.696	<0.001
Malignancy	1.775	1.629-1.934	<0.001
Cerebrovascular disease	0.866	0.812-0.925	<0.001
Steroids or immunosuppressants	1.403	1.281-1.537	<0.001
Use of carbapenem	1.487	1.343-1.645	<0.001
Oxygen administration	1.871	1.750-1.999	<0.001
Mechanical ventilation	1.663	1.458-1.898	<0.001

Abbreviations: BMI, body mass index; CI, confidence interval; COPD, chronic obstructive pulmonary disease.

Table S2. Multivariate analysis of factors associated with in-hospital mortality: use of non-carbapenem antipseudomonal antibiotics

	Odds ratio	95% CI	*p* value
Age (years)	1.027	1.023-1.031	<0.001
Sex (male)	1.745	1.642-1.854	<0.001
BMI (kg/m^2)	0.911	0.903-0.918	<0.001
Barthel index	0.987	0.986-0.989	<0.001
Current/past smoker	0.974	0.910-1.043	0.455
Coma	1.695	1.518-1.893	<0.001
Emergency transport	0.954	0.902-1.009	0.101
Hugh-jones classification	0.989	0.976-1.002	0.093
Chronic heart failure	1.403	1.309-1.504	<0.001
Dementia	0.867	0.817-0.921	<0.001
Interstitial pneumonia	2.047	1.673-2.505	<0.001
Asthma	0.692	0.584-0.821	<0.001
COPD	1.339	1.093-1.641	0.005
Bronchiectasis	1.526	1.182-1.970	0.001
Renal failure	1.544	1.393-1.711	<0.001
Malignancy	1.769	1.623-1.928	<0.001
Cerebrovascular disease	0.866	0.811-0.924	<0.001
Steroids or immunosuppressants	1.416	1.293-1.551	<0.001
Use of non-carbapenem antipseudomonal antibiotics	1.217	1.146-1.292	<0.001
Oxygen administration	1.874	1.754-2.003	<0.001
Mechanical ventilation	1.718	1.507-1.959	<0.001

Abbreviations: BMI, body mass index; CI, confidence interval; COPD, chronic obstructive pulmonary disease.
